# Anatomical variant of the meniscus related to posterior junction: a case report

**DOI:** 10.1186/s13256-017-1512-z

**Published:** 2017-12-18

**Authors:** David Sadigursky, Lucas Cortizo Garcia, Rodrigo Rêgo Martins, Gustavo Castro De Queiroz, Rogério Jamil Fernandes Carneiro, Paulo Oliveira Colavolpe

**Affiliations:** 1 Division of Knee Surgery, Clínica Ortopédica Traumatológica – COT, Salvador, Bahia Brazil; 2 Section of Medicine, Faculdade de Tecnologia e Ciências - FTC, Salvador, Bahia Brazil

**Keywords:** Meniscus, Knee, Congenital, Anterior cruciate ligament

## Abstract

**Background:**

There are several reports on anatomical differences of the meniscus. However, there are only a few reports on abnormalities in both menisci and anatomical differences in anterior cruciate ligament insertions.

**Case presentation:**

This is a case report of a 36-year-old Hispanic man presenting symptoms, including knee pain, locking, and effusion, with an anatomical abnormality of the menisci corresponding to the fusion of the posterior horns of the menisci in tandem with the insertion of the posterior meniscus fibers in the anterior cruciate ligament.

**Conclusions:**

This is the first study describing a meniscus anatomical variant with isolated posterior junction of the posterior horn with an anomalous insertion to the anterior cruciate ligament. The recognition of meniscus variants is important as they can be misinterpreted for more significant pathology on magnetic resonance images.

## Background

Anatomical anomalies are commonly reported because of their importance in the development of conditions limiting daily physical activities. In addition, it is notable that the human meniscus presents with different kinds of anatomical abnormalities [1]; those frequently observed are a discoid meniscus (DM) and abnormalities of the meniscal horns [2]. Other malformations, including ring-shaped meniscus, meniscal ossicle, and insertional abnormality, are rare [3]. To the best of our knowledge, there are no reports on the fusion of meniscal posterior horns in tandem with the insertion of meniscal posterior fibers in the anterior cruciate ligament (ACL).

We report the first case of symptomatic anatomical meniscus alteration, presenting with a ring-shaped lateral meniscus concomitant with a fusion of posterior contours of menisci and insertion of meniscal posterior fibers in the ACL in a 36-year-old Hispanic man.

In the present case report, the patient’s consent was obtained by the patient signing the Terms of Consent. The approval of the Research Ethics Committee of the institution was also obtained. Data were collected between April 02 (2015) and August 28 (2015), the date of the patient’s last visit. A review of articles on the subject was conducted using the following scientific platforms: PubMed, SciELO, Science Direct, Medline, and Google Scholar. The following keywords were used for the search: “knee pain” and/or “meniscal anatomic variants” and/or “meniscus anomaly” and/or “meniscus anatomy” and/or “meniscus abnormality.”

## Case presentation

A 36-year-old Hispanic man presented at our medical center with pain in his right knee of approximately 6 months’ duration, along with knee locking and effusion. There was no history of trauma.

During a physical examination, he was in good health with no comorbidities or congenital diseases. During an orthopedic examination of his right knee, the range of motion was found to be preserved (0 to 155°), with joint crepitation, and positive Steinmann and Merke tests results [4]. McMurray test result was negative; ligament tests were normal and there was no patellofemoral joint pain [5].

**Fig. 1:**
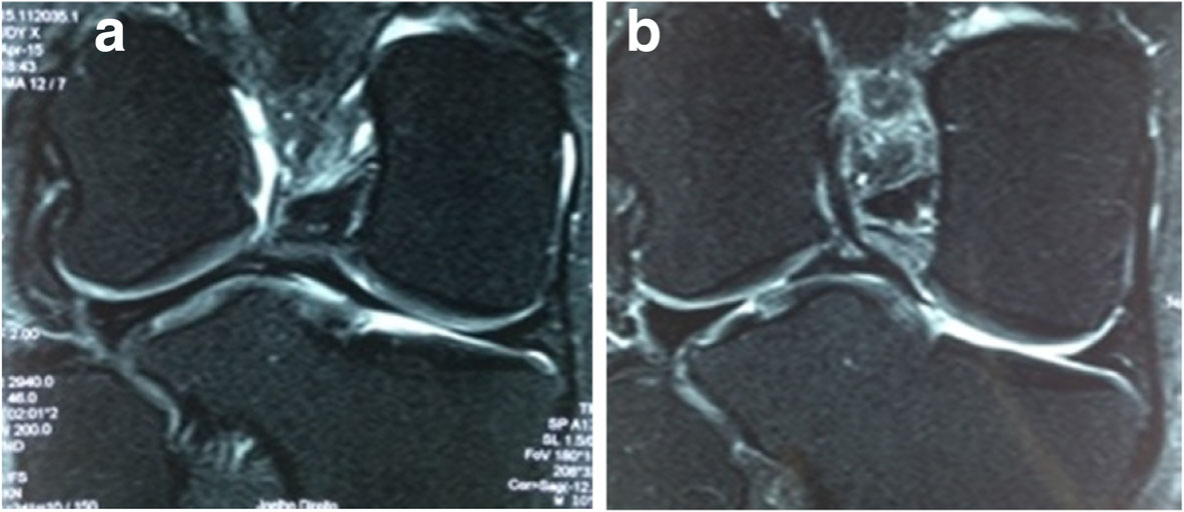
**a** and **b** Magnetic resonance imaging in coronal T2 showing the meniscal variant of posterior junction and insertion to the anterior cruciate ligament

**Fig. 2:**
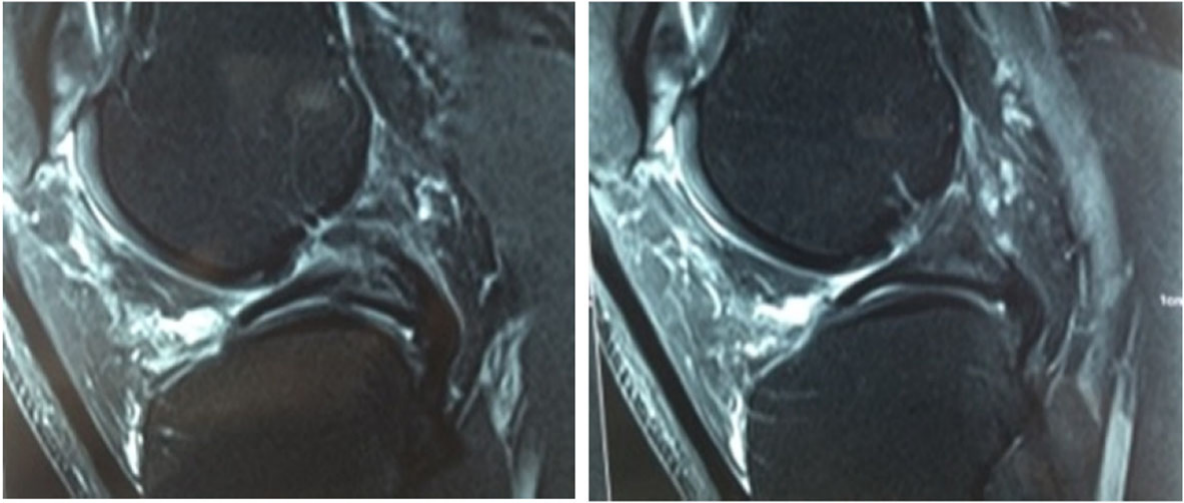
Magnetic resonance imaging in sagittal T2 demonstrating the meniscal variant of posterior junction and insertion to the anterior cruciate ligament

Magnetic resonance imaging (MRI), performed on 17 April 2015, revealed the following findings: a ring-shaped lateral meniscus, with small spots of radial rupture, adjacent to the free margin; degenerative changes in medial meniscus, emphasizing that there was a fusion of posterior contours of the menisci characterized as an anatomical variation; visible thinning of the ACL, including a part of its fibers from the posterolateral segment inserted in the posterior horn of the lateral meniscus (constitutional aspect; Figs. 1 and 2).

**Fig. 3:**
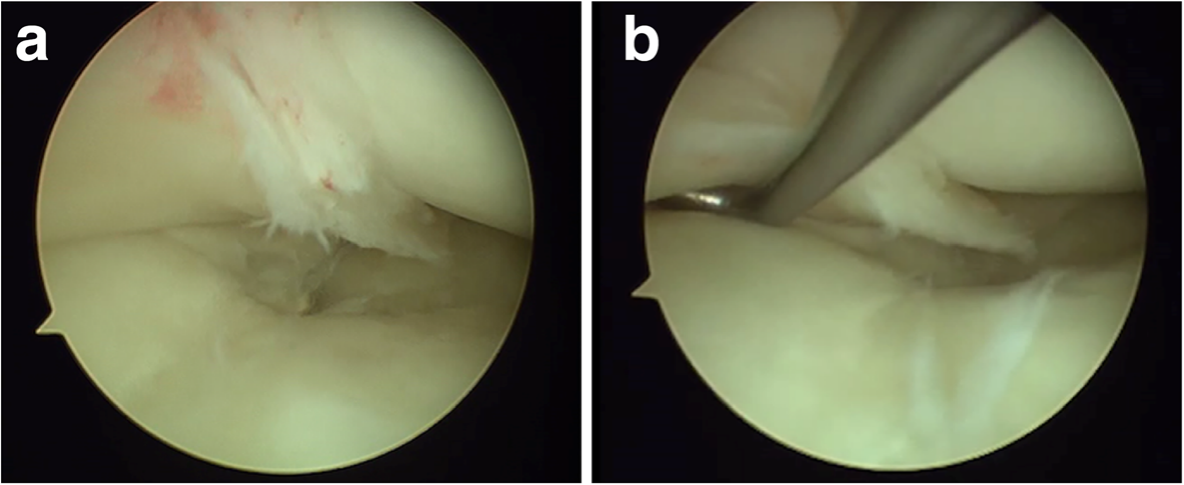
**a** and **b** Arthroscopic images of the complete posterior junction of the posterior horns of the menisci

Our patient was initially treated using a conservative approach and a physiotherapy program. Because of persistent pain with mechanical blocking of maximum flexion, surgical treatment was indicated, with arthroscopy of his right knee, which was performed 4 months after the initial treatment began. During arthroscopic inspection, the following findings were highlighted: first, lateral meniscus with degenerative lesion in the posterior horn; and second, anatomical changes, that is, junction of both lateral and medial menisci posterior horn with interposition in the medial femoral condyle and insertion of meniscal fibers in the ACL (Fig. 3) were considered the cause of knee locking and of the presence of audible clicks.

A partial meniscectomy was performed, preserving the meniscal roots and decreasing the menisci interposition in the femorotibial joints, regulating the anatomical variation.

He developed no postoperative pain and the results were satisfactory.

## Discussion

The present case describes a 36-year-old man who sought a consultation at an orthopedics hospital. He complained of knee pain associated with clamping and crepitation, common symptoms during every day clinical practice, except that, in this case, he had a finding of anatomical variation of the menisci on MRI showing fusion of posterior horns of the menisci and posterior insertion to the ACL.

Based on the normal anatomy of the menisci, they can be divided into three parts: body, anterior horn, and posterior horn. The function of both anterior and posterior horns is to secure the menisci in the tibia’s plateau; these are critical structures for its biomechanical function [6, 7]. Lesions in this region cause a loss in menisci biomechanical function, leading to an early degeneration of the articular cartilage, and, thereby, causing osteoarthritis [8, 9].

Among the morphological variations of the menisci, the most common is the DM, which probably has an embryological etiology. In DM, the meniscus shape resembles a disk instead of the usual shape (with an ascending shape), with greater incidence on the lateral meniscus (77%) [10, 11]. Its clinical presentation is variable, from asymptomatic to the presence of pain, crepitation, and decreased range of motion, especially in children and young adults [6, 12]. With the exception of the lateral meniscus, other malformations are infrequent, having a total incidence of 0.3% [13]. These anatomical changes may present as a meniscus with a ringed shape, having two layers, meniscal ossicle, or insertional abnormalities, such as anomalous insertion of the meniscus posterior horn into the ACL [3, 14].

The ring-shaped meniscus presents a circular form, with its external part being similar to the one of a normal meniscus: well-defined and angular, with no inner portion mobility, near the intercondylar notch. There is also a description of the meniscal ossicle, which is an uncommon alteration that normally occurs in the posterior horn of medial meniscus; it is defined as the presence of cortical and trabecular bone with bone marrow surrounded by meniscal fibrocartilage [15].

A study on the anatomy of the menisci anterior horns points to a change of its insertion in the ACL, identified in 35 knees from cadavers of Ghana’s population [16]. The anomalous insertion of the medial meniscus anterior horn in the ACL may also occur in the posterior horn of the medial or lateral meniscus [17–19]. Other changes are much less frequent, with an incidence of 0.3% [13]. They are more frequent in the Asian population, and the lateral meniscus is the most affected. Mostly, these changes are asymptomatic [1, 20].

The meniscal variation presented in this study was not described in any other study on meniscal abnormalities. Additional investigation of similar cases is required so that a suitable description can be added among the possible anatomical variations of the menisci.

## Conclusions

This is the first study describing a meniscus anatomical variant with isolated posterior junction of the posterior horn with an anomalous insertion to the ACL. The recognition of meniscus variants is important as they can be misinterpreted for more significant pathology on magnetic resonance images.

## Abbreviations

ACL: Anterior Cruciate Ligament
DM: Discoid meniscus
MRI: Magnetic resonance imaging
